# Caffeine Treatment Prevented from Weight Regain after Calorie Shifting Diet Induced Weight Loss

**Published:** 2014

**Authors:** Sayed Hossein Davoodi, Seyed Javad Hajimiresmaiel, Marjan Ajami, Anoushiravan Mohseni-Bandpei, Seyyed Abdulmajid Ayatollahi, Kamran Dowlatshahi, Gholamali Javedan, Hamidreza Pazoki-Toroudi

**Affiliations:** a *National Nutrition and Food Technology Research Institute, Faculty of Nutrition and Food Technology, Shahid Beheshti University of Medical Sciences, Tehran, Iran. *; b *Department of Cardiology, Iran University of Medical Sciences. *; c *Department of Food and Nutrition Policy and Planning Research, National Nutrition and Food Technology Research Institute, Faculty of Nutrition and Food Technology, Shahid Beheshti University of Medical Sciences, Tehran, Iran. *; d *School of Health, Shahid Beheshti University of Medical Sciences. *; e *Department of Pharmacognosy, Phytochemistry Research Center, School of Pharmacy, Shahid Beheshti University of Medical Sciences, Tehran, Iran.*; f *Islamic Azad University, Najafabad Branch, Isfahan, Iran. *; g *Department of Nutrition, Iran University of Medical Sciences. *; h *Physiology Research Center. Iran University of Medical Sciences.*

**Keywords:** Caffeine, Calorie shifting diet, Diet, Obesity, Resting metabolic rate, Weight loss

## Abstract

Low calorie diets are always difficult for obese subjects to follow and lead to metabolic and behavioral adaptation. Therefore, we evaluated the effect of caffeine treatment with calorie shifting diet (CSD) on weight loss.

Female subjects (n=60; BMI≥25) completed 4-weeks control diet, 6-weeks CSD (3 repeated phases; each 2-weeks) and 4-weeks follow-up diet, with or without caffeine treatment (5 mg/Kg/day). The first 11 days of each phase included calorie restriction with four meals every day and 4 hours intervals.

Significant weight and fat loss were observed after 4-weeks of CSD (5.7 ± 1.24 Kg and 4.84 ± 1.53 Kg) or CSD+Caffeine (7.57 ± 2.33 Kg and 5.24 ± 2.07 Kg) which was consistent for one month of the follow-up (CSD: 5.24 ± 1.83 Kg and 4.3 ± 1.62 Kg, CSD+Caffeine: 12.11 ± 2.31 Kg and 9.85 ± 1.6 Kg, p < 0.05 vs CSD group) and correlated to the restricted energy intake (p < 0.05). During three CSD phases, RMR tended to remain unchanged in both groups.While, CSD or CSD + Caffeine treatments, significantly decreased plasma glucose, total-cholesterol, and triacylglycerol (p < 0.05), even during follow-up period (p < 0.05). HDL-cholesterol was not changed by CSD. Feeling of hunger decreased and subject’s satisfaction increased after 4-weeks of CSD (p < 0.05) and remained low to the end of study, while satiety was not affected. Coffeine increased the effect of CSD on feeling of hunger and subject’s satisfaction after week 7 (p < 0.05 vs. CSD).

These findings indicated that combination of caffeine treatment with CSD could be an effective alternative approach to weight and fat loss with small changes in RMR and improved tolerance of subjects to the new diet.

## Introduction

Obesity is defined as abnormal or excessive fat accumulation that may impair health and in the past decade, has become a global problem especially in developed countries. With more than 1.6 billion overweight adults over the age of 20, obesity has a number of serious consequences for individuals and government health system (1). World Health Organization notes that obesity is the fifth leading risk factor for death globally. At least 2.8 million adults die each year as a result of being overweight. Such increase in the prevalence of obesity is the result of two important factors; increased intake of energy-dense foods and decrease in physical activity (2, 3). Resting metabolic rate (RMR), physical activity and thermic effect of food are three major components of daily energy expenditure (4, 5). 

Obesity increases the risk of morbidity and mortality and reduces quality of life and weight loss is a primary treatment strategy for obesity reduction (6, 7). Reduced metabolism after a period of weight loss may result in positive energy balance and relapse of obesity in many people (8).

Calorie restriction may produce weight loss over short term but is hard for obese patients to follow because food intake must be limited every day (9-12). To overcome this situation we examined a new dieting strategy, calorie shifting diet, in which the body receives different calories instead of consistent low-calorie diets to trick the body into thinking that there has been no or little change in calorie intake, therefore avoiding the body’s tendency to trigger survival mode and so preventing the adaptation to new conditions by confusing the metabolic system (12). 

It has been demonstrated that supplementary diet including caffeine can decrease the weight through the increase in thermogenesis and fat oxidation (13-15). Caffeine, probably increase thermogenesis and fat oxidation through inhibition of phosphodiesterase, which degrades intracellular cyclic AMP, and inhibition of negative effects of adenosine on increased noradrenaline release (16). Therefore present study tried to evaluate the effect of CSD and CSD+Caffeine on weight loss treatment and metabolism status.

## Experimental

*Ethics statement*

This study was done in accordance to the declarations of Helsinki, and was approved by the ethic committee of Shahid Beheshti University of Medical Sciences, Tehran, Iran. All participants received required information about study and after signing written consent, were introduced into the study. 

*Subject selection*

The subjects were 60 overweight or obese [body mass index (BMI) Kg/m^2^: 25–39.9 for CSD (n=30) and 30 for CSD + Caffeine group] but otherwise healthy female aged between 26-45 y with sedentary life style, who were selected from two clinics related to Weight Loss and Weight Gain Unit, Shohaday Tajrish Hospital and Private Clinic of Dr Dowlatshahi, between April 2010 and September 2011. Ninety nine female subjects were screened for the study; from which 39 were excluded at the first step because of being out side of inclusion criteria. All subjects gave written informed consent before participating and were provided with a stipend. Required nutritional instructions were provided by a registered dietitian. Demographic data and baseline variables are summarized at Table 1. 

**Table 1:** Physical characteristics of subjects

		**Basal**	**Calorie Shifting Diet (CSD)**						**Follow-up**
		Day 0	**Phase 1** Day 1-11	Day 12- 14	**Phase 2** Day 15-25	Day 26-28	**Phase 3** Day 29-39	Day 40-42	Day 43-70
CSD	Age (y)	**38.87 ± 6.27**							
	Height (cm)	**166.35 ± 12.24**							
	Weight (Kg)	**83.06 ± 2.11**	**80.94 ± 2.63**	81.27 ± 1.93	77.04 ± 1.96 *	77.36 ± 1.9 *	**76.21 ± 1.83 ** **	**77.08 ± 1.66 ** *	**77.82 ± 1.92 ** *
	Weight loss (%)	**--------**	**2.55 ± 0.2**	2.15 ± 0.18	6.81 ± 0.40 *	8.24 ± 0.31 *	**8.25 ± 0.43 ** *	**7.19 ± 0.27 ** *	**6.30± 0.39 ** *
	Body total fat (kg)	**36.48 ± 2.11**	**34.61 ± 1.86**	34.5 ± 1.97	**32.33 ± 1.13 ** *	31.64 ± 1.69 *	**31.25 ± 2.01 ** *	**31.4 ± 2.14 ** *	**32.18 ± 2.3 ** *
	Fat loss (%)	**--------**	**5.12 ± 2.81**	5.42 ± 2.76	**11.37 ± 3.22 ** *	13.26 ± 2.97 **	**14.35 ± 3.71 ** **	**13.92 ± 3.59 ** **	**11.78 ± 3.38 ** *
	BMI (Kg/m^2^)	**30.14 ± 1.12**	**29.37 ± 0.56**	29.49 ± 1.6	**27.95 ± 0.91 ** *	28.07 ± 1.0 4	**27.65 ± 1.27 ** *	**27.97 ± 0.8 ** *	**28.24 ± 0.71 ** *
	WHR	**0.93 ± 0.02**	**0.91 ± 0.02**	0.90 ± 0.03	**0.87 ± 0.02 ** *	0.87 ± 0.02 *	**0.85 ± 0.02 ** *	**0.86 ± 0.03 ** *	**0.88 ± 0.02 ** *
CSD + Caffeine	Age (y)	**39.58 ± 5.36**							
	Height (cm)	**168.28 ± 14.32**							
	Weight (Kg)	**85.12 ± 3.24**	**83.25 ± 3.04**	82.47 ± 2.51	**79.91 ± 2.24 ** *	77.55 ± 2.41 *	**76.60 ± 2.50 ** **	**75.11 ± 2.17 ** *	**73.01 ± 2.11 ** *
	Weight loss (%)	**--------**	**2.19 ± 0.4**	3.11 ± 0.38	**6.12 ± 0.67 ** *	8.89 ± 0.81 *	**10.0 ± 0.81 ** *	**11.75 ± 1.27 ** *	**14.22 ± 1.59 ** * ‡
	Body total fat (Kg)	**39.76 ± 3.27**	**37.58 ± 2.33**	37.6 ± 2.61	**36.47 ± 2.80 ** *	34.52 ± 2.29 *	**32.15 ± 2.71 ** *	**31.72 ± 2.54 ** *	**30.18 ± 3.4 ** *
	Fat loss (%)	**--------**	**5.48 ± 2.81**	5.74 ± 2.76	**8.75 ± 3.22 ** *	14.36 ± 2.97 **	**22.04 ± 3.71 ** **	**25.0 ± 3.59 ** **	**30.2 ± 3.38 ** * ‡
	BMI (Kg/m^2^)	**30.15 ± 1.32**	**29.49 ± 0.94**	29.21 ± 0.87	**28.31 ± 0.89 **	27.47 ± 0.64 *	**27.14 ± 0.82 ** *	**26.61 ± 0.84 ** *	**25.86 ± 0.61 ** * ‡
	WHR	**0.94 ± 0.02**	**0.93 ± 0.02**	0.91 ± 0.03	**0.89 ± 0.02 ** *	0.86 ± 0.02 *	**0.84 ± 0.02 ** *	**0.83 ± 0.03 ** *	**0.84 ± 0.02 ** * ‡

All data expressed as Mean ± SEM. Data of basal value show the mean of all obtained data from subjects during one month of control period. During CSD and follow-up period data were obtained at the end of each stage (*e.g*. end of day 11 at phase 1. WHR: Waist to Hip Ratio. The percent of weight loss and fat loss calculated from the amount of loss regarded to the baseline value.

* p < 0.05 and

** p < 0.01 vs. baseline value (One way ANOVA with post hoc Tukey test), and

‡ p < 0.05 vs. value of same date for CSD group in comparison to the CSD+Caffeine group (Student t-test).

*Exclusion criteria*

Pregnant, lactating or post-menopausal women were excluded. Also subjects with diabetes, cardiovascular disease, eating disorders, psychological disorders, substance abuse or regular use of medications except contraceptives were excluded. Patients who participated in an exercise class or took weight loss, lipid or glucose lowering medications and whose activity for 3 months prior to the study was more than light (*i.e*. > 3 h/week of light intensity exercise at 2.5 to 4.0 metabolic equivalents) were also excluded.

*Diets and treatments*

Calorie shifting diet (CSD) regimens include 3 phases; each phase consists of fast days (energy restricted diet) for 11 days and feed days (ad libitum food intake) for 3 days. Consumption of calories on fast days was restricted to 1350. 

In order to obtain information about their diet patterns and routine daily calorie intake subjects filled a (FFQ) for one month and a recall questionnaire for last 5 days. The basal calorie intake for every subject was calculated as the mean of calorie intakes per day during last month and used as a starting point to calculate required calorie intake for the present study (For example for a subject with mean daily intake of 2000 kcal for last the month, the new regimen included 1200 kcal/day and for a subject with 2300 kcal for last month new regimen included 1500 kcal/day). All subjects were instructed to consume their meals (containing determined calorie) only at 4 set of meals every day and avoid any other intake at other times of day. The time for each of these meals was optional and they were free to consume in any hour, but the time interval between meals could not be less than 4 hours (*e.g*. 8-12-4-8). This diet was ordered for 11 consecutive days after which 3 days of self-selecting calorie and diet was started. The 11-3 days phases were repeated 3 times (for 42 days) and finally a month fallow-up period was started in which subjects received weight maintenance calorie intake. 

All subjects were randomly divided in two groups; The first group just received CSD and the second group received CSD + Caffeine (oral solution 5 mg/Kg/day/250 mL in water from beginning of study to the last day of evaluation) 

*Assessments*

At the beginning of study (before diet treatment) body weight was measured on an electronic weighing scale (Mettler Toledo IDL Plus, Eichfahig, Germany), with nearest to the 0.25 Kg, in light clothing. Height was measured using a stadiometer calibrated before each measurement (Holtain *Ltd*., Crymych, UK). Abdominal and hip circumferences were assessed to the nearest 0.1 cm by research dietitians. *BMI*: Body mass index was calculated as [weight (Kg)] **/ **[height (m)]^2^. The waist to hip ratio (WHR) was determined from waist (minimal circumference of the abdomen) and hip (greatest circumference of the greater trochanters) circumferences measured to the nearest 1.0 cm. 

*Body fat mass and fat mass percent*

Body composition was measured after 20 minutes of rest, using bioelectrical impedance analysis (BIA 101, Akern Bioresearch, Florence, Italy). After cleaning the skin with alcohol, two electrodes were placed on the surface of the right hand and right foot. The low intensity (800 mA) current with 50 KHz frequency was delivered. Guidelines of National Institutes of Health (17), and method of Lukaski used to perform measurements (8) which included fat free mass (FFM), fat mass (FM) and FM percentage as (FM/body weight) × 100. 

*RMR: *Metabolic test was done at baseline, at the morning of each day 11 hours after the last meal. RMR was measured at days 0, 11, 14, 25, 28, 39, 42 and 72 of the study. RMR was measured over a 60-minute period by indirect calorimetry using a Deltatrac II Metabolic cart (Datex-Ohmeda, Helsinki, Finland). After resting calmly for 20 minutes and reaching a rest condition, a transparent plastic hood connected to the device was placed over the head of the participant. Constant air flow (40 L/min) generated by the metabolic system was used to dilute inspired and expired air. Computations of O_2_ utilization and CO_2_ production were done from constant measurements of O_2_ and CO_2_ concentrations to determine RMR. Subjects were instructed to remain motionless and awake during the test, and RMR was calculated by utilizing data which was obtained from the last 30 minutes of the measurement.

Blood samples were collected for the measurement of lipids and glucose. Plasma was isolated by centrifugation at 2000×g for 10 min at 5 °C and frozen at -20 °C. Biochemical analysis was performed at the end of the study for all samples. Plasma glucose, total cholesterol, triacylglycerol and HDL-cholesterol concentrations were measured with a Cobas Bio-centrifugal-analyzer (Roche). 

*Visual analog scale (VAS)*

Subjects completed a validated VAS at the baseline and at the end of each week of study. In summary, the VAS consisted of 100 mm lines, and subjects were asked to make a vertical mark across the line corresponding to their feelings from 0 (not at all) to 100 (extremely) for hunger, satisfaction with diet, or fullness. 

*Statistical analysis*

All data were expressed as Mean ± SEM (Standard error of the mean). The main variables tested included body weight, body fat, Waist to hip ratio, BMI, RMR, nutrient intake, blood parameters, hunger, satisfaction and fullness. ANOVA performed to determine p-value over time and then Post Hoc Tukey test was performed to assess significance. P ≤ 0.05 was considered significant. For each week of study the results were compared with baseline value. Analyses were run with SPSS 14.0 for Windows (SPSS Inc, Chicago, IL). Two Subject withdrew during the study at week 2 and 5 of CSD or CSD + Caffeine treatment for personal reasons. Data is therefore presented for subjects. Difference between groups was analyzed by student t-test. Relations between continuous variables (*i.e.* body weight, fat mass, BMI, RMR, nutrient intake, hunger, satisfaction and fullness) were assessed by simple regression analyses as appropriate. 

## Results

The mean age of participants was 38.87 ± 6.27 years in CSD group and 39.58 ± 5.36 years in CSD+Caffeine group. Patients’ characteristics are presented in Table 1.

*Changes in body weight, fat mass and BMI in response to diet or treatment*

*Weight loss*

The mean value of basal weight was 83.06 ± 2.11 Kg and 85.12 ± 3.24 for CSD and CSD + Caffeine groups, and the changes over one month of control period was not significant (data related to one month control period was not included in results except the mean values for baseline). There was no significant difference compared to baseline at day 11 (phase 1 CSD) and next 3 days of self-selecting diet for both groups (Table 1), however at the end of day 25 (phase 2 CSD) total weight loss of 6.02 ± 1.2 and 5.21 ± 2.31 were attained for CSD and CSD+Caffeine groups (p < 0.05 and p < 0.05 vs. baseline for each group, respectively) which remained unchanged for the next 3 days (Table 1). More decrease was achieved at the end of phase 3 CSD (day 39; p < 0.01) that remained unchanged for 3 consecutive days of optional calorie intake. In CSD group, after one month of weight maintenance, calorie intake only increased 0.74 Kg in body weight (p < 0.01 vs. baseline and p=0.26 vs. day 39) while in the CSD + Caffeine treated group the weight tended to decrease further (P < 0.05 vs CSD, day 70 Table 1). 

*Body fat and WHR*

Total body fat decreased significantly after phase 2 and phase 3 of both treatments (p < 0.05). Percent of fat loss from baseline value was also decreased at phases 2 and 3 and follow-up period (p < 0.01). However, the decrease in the CSD + Caffeine group was significantly more than CSD group (p < 0.05, Table 1). The baseline value of waist to hip ratio was 0.93 ± 0.02 and 0.94 ± 0.02 in CSD and CSD + Caffeine groups, which significantly decreased in both groups (p < 0.05), except the first phase.

*BMI*

Body mass index was affected by CSD or CSD+Caffeine regimens and decreased from baseline value of 30.02 ± 1.12 Kg/m^2^ or 30.15 ± 1.32 Kg/m^2^ to 27.84 ± 0.91 Kg/m^2^ and 27.47 ± 0.64 at the end of phase 2 , and 27.54 ± 0.8 Kg/m^2^ and 26.61 ± 0.84 at the end of phase 2, respectively (Table 1). Similar to the results obtained from weight loss, both kind of treatments caused significant decrease in BMI at phase 2 (day 25; p < 0.05) and phase 3 (day 39; p < 0.05) which remained unchanged during 3 days intervals of self-selecting diet and 30 days of follow-up period, when weight maintenance calorie intake was prescribed (p < 0.05). However, the effect of CSD + Caffeine was more significant than the CSD (p < 0.05, day 70; Table 1).

*Energy intake*

Energy intake and energy restriction were determined from food record data collected in each day of study at baseline, CSD and follow-up period which are summarized at Table 2. During one month of control phase, mean energy intake was 2462 ± 257 kcal, that decreased to 1352 ± 254 (p < 0.01) during 11 days of CSD, 2021 ± 242 (p < 0.05) at 3 days interval and 1606 ± 260 (p < 0.05) at follow-up period. Degree of energy restriction due to CSD was correlated to the rate of weight loss in both CSD and CSD + Caffeine groups (r = 0.46, P < 0.05 and r = 0.43, p < 0.05, respectively). The percents of calorie intake in the form of carbohydrates remained unchanged during the CSD phases and follow-up period, while the percents of calorie intake in the form of fats decreased during the CSD phases and weight maintenance period. 

**Table 2:** Composition of diets

			**Baseline**	**11 days CSD**	**3 days intervals**	**Follow-up**
**Energy**	(kcal/d)	2462 ± 257	1352 ± 254 **	2021 ± 242 *	1606 ± 260 *
**Carbohydrate **	%	55	55	55	55
	(g/d)	338	185	277	220
	(g/meal)	67.6	46	55.4	55
	(cal/day)	1354	744	1112	883
	(cal/meal)	270.8	186	222.4	220.8
				
	%	15	25	20	20
**Protein**	(g/d)	92	84	101	80
	(g/meal)	18.4	21	20.2	20
	(cal/day)	369	338	404	321
	(cal/meal)	73.8	84.5	80.8	80.3
				
	%	30	20	25	25
**Fat**	(g/d)	82	30	56	44
	(g/meal)	16.4	7.5	11.2	11
	(cal/day)	739	270	505	402
	(cal/meal)	147.8	67.5	101	100.5

Data is expressed as the mean of values obtained during specific stage of study. Data about CSD and 3 days intervals are the mean of three phases of CSD period.

* p<0.05 and

** p<0.01 vs. basal level. (Analyzed by: one way ANOVA and post hoc Tukey test).

*Changes in RMR in response to CSD*

**Figure 1:**
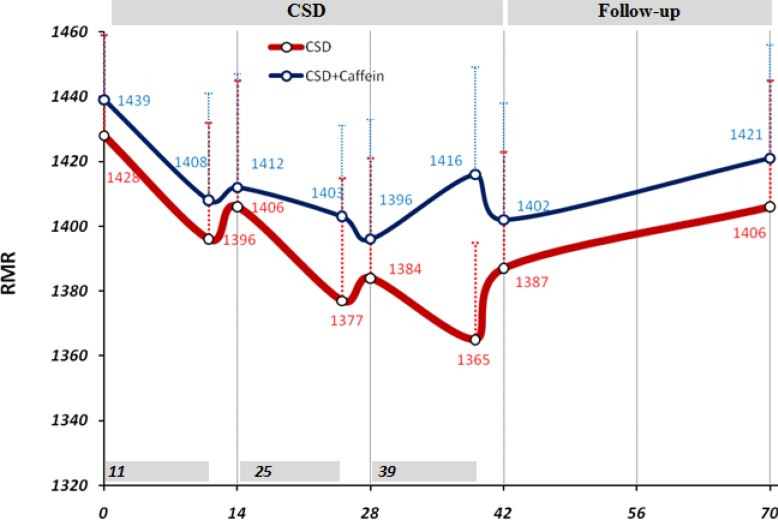
Changes in resting metabolic rate (RMR) due to CSD regimen. 14 days (2-weeks) used as minor units of x-axis while the data of 11 days CSD also has been shown just before each 14 days. Two way ANOVA was applied to analysis the differences between RMR values of same group in mentioned date with the baseline value, and student t-test applied to compare between caffeine treated and untreated groups in the same dates. There was no significant difference between groups

Before the initiation of CSD regimen the mean value of RMR, extracted from data of one month ago, was equal to 1428 ± 31 kcal/day and 1439 ±29 kcal/day for CSD and CSD + Caffeine groups. Although during the CSD phase 1, 2 and 3 the levels of RMR tended to decrease to in both groups, the difference with baseline RMR level was not statistically significant (Figure 1). During 4 weeks of follow-up period RMR returned to levels close to the basal value (1406 ± 39 kcal/day and 1421 ± 35). 

*Changes in Biochemical factors in response to CSD*

Table 3 summarizes the values for fasting plasma glucose, total-cholesterol, HDL-cholesterol, and triacylglycerol concentrations before and after dietary intervention. These evaluations showed that CSD or CSD + Caffeine regimens after 39 days significantly decreased plasma glucose, total-cholesterol, and triacylglycerol levels (p < 0.05 vs. baseline values). Similar decrease was seen after 3 days of self-selecting diet at day 42 (p < 0.05). After 4 weeks of maintenance regimen during follow-up period, the levels of plasma glucose and total-cholesterol remained at significantly low levels compared to baseline value (p < 0.05), while triacylglycerol levels returned to levels near the baseline values (p = 0.1). There was no significant difference in levels of plasma HDL-colestrol between different stages of study (Table 3). 

**Table 3:** Biochemical factors before and after treatment in plasma

		**Baseline day 0**	**CSD day 39**	**Day 42**	**Follow-up day 70**
**CSD**	**Glucose** (mmol/L)	5.59 ± 0.34	4.31 ± 0.29 *	4.62 ± 0.30 *	4.77 ± 0.32 *
	**Total** **cholesterol** (mmol/L)	5.53 ± 0.39	4.16 ± 0.41 *	4.70 ± 0.41 *	4.75 ± 0.33 *
	**HDL-colestrol** (mmol/L)	0.99 ± 0.12	1.22 ± 0.14	1.01 ± 0.14	1.17 ± 0.19
	**Triacylglycerol** (mmol/L)	2.62 ± 0.33	1.57 ± 0.42 *	1.77 ± 0.31 *	2.09 ± 0.44
**CSD + Caffeine**	**Glucose** (mmol/L)	5.58 ± 0.33	4.17 ± 0.37 *	4.26 ± 0.39 *	4.18 ± 0.32 *
	**Total** **cholesterol** (mmol/L)	5.40 ± 0.38	4. 23± 0.31 *	4.52 ± 0.30 *	4.48 ± 0.32 *
	**HDL-colestrol** (mmol/L)	0.86 ± 0.11	0.91 ± 0.14	0.97 ± 0.16	1.07 ± 0.22 *
	**Triacylglycerol** (mmol/L)	2.58 ± 0.29	1.80 ± 0.31 *	1.63 ± 0.28 *	1.84 ± 0.34 *

Data were expressed as Mean ± SEM.

* p<0.05 vs. baseline value (One way ANOVA with post hoc Tukey test). There was no significant difference between CSD and CSD+Caffeine groups.

*VAS evaluations of the effects of CSD on subjects Hunger, satisfaction and fullness*

**Figure 2:**
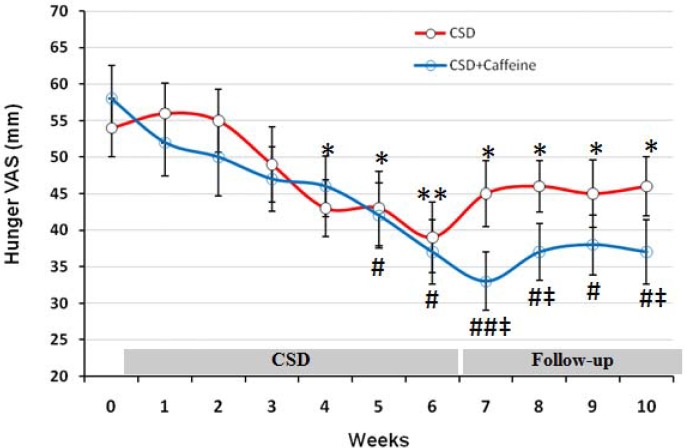
The VAS for hunger during each stage of study at CSD system. The graph shows the mean values which was obtained at the end of each week of study. From week 0 to 6 belongs to the CSD and from week 7 to 10 belongs to follow-up periods. * p<0.05 and ** p <0.01 vs. baseline value of CSD group, # p<0.05 and ## p <0.01 vs. baseline value of CSD + Caffeine group (Two way ANOVA with post hoc Tukey test) and ‡ p<0.05 vs. value of same day for CSD group in comparison to the CSD+Caffeine group (Student t-test).

**Figure 3:**
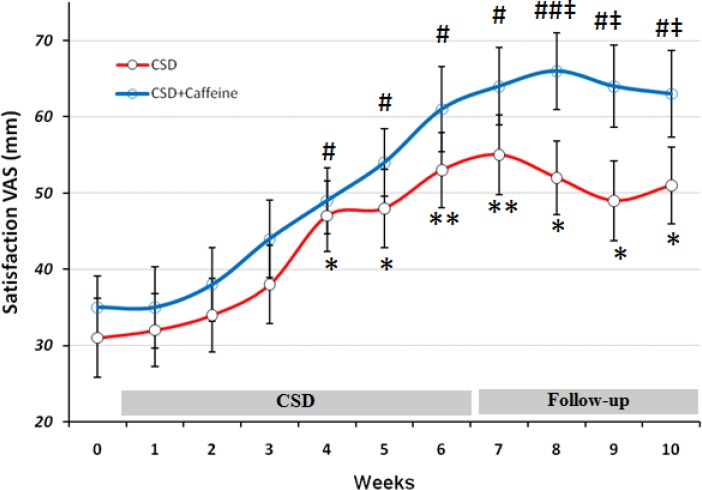
The VAS for Satisfaction during each stage of study at CSD system. The graph shows the mean values which was obtained at the end of each week of study. *p < 0.05 and **p < 0.01 vs. baseline value of CSD group, # p < 0.05 and ## p < 0.01 vs. baseline value of CSD + Caffeine group (Two way ANOVA with post hoc Tukey test) and ‡ p<0.05 vs. value of same day for CSD group in comparison to the CSD+Caffeine group (Student t-test).

**Figure 4:**
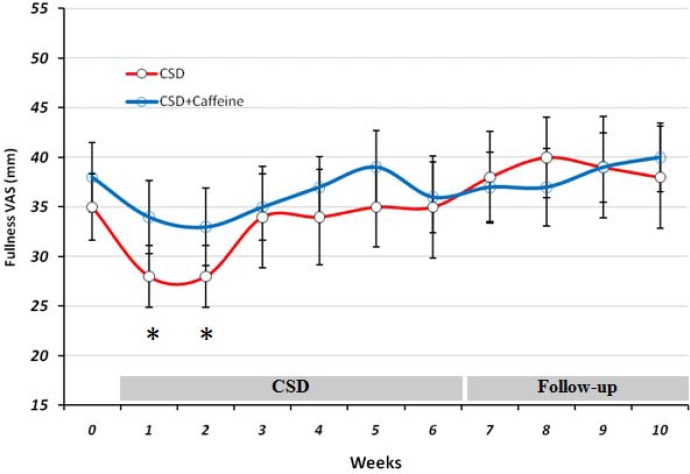
The VAS for Fullness during each stage of study at CSD system. The graph shows the mean of values which obtained at the end of each week of study. *p < 0.05 vs. baseline value of CSD group(Two way ANOVA with post hoc Tokey test).

Changes in hunger, satisfaction, and fullness were evaluated by visual analog scale at baseline and each day during the trial and are shown as mean for each week of study in Figures 2 to 4. The baseline feeling of hunger was marked as 54 mm by participant subjects, which non-significantly increased during the first and second weeks of CSD (56 and 55 mm, respectively; Figure 1). However from week 3 of CSD it started to decrease and at weeks 4 to 6 of CSD it was significantly less than baseline value (p < 0.05 for weeks 4 and 5 and p < 0.01 for week 6). During follow-up period the hunger scores increased mildly but was still less than baseline values (p < 0.05). The effect of CSD+Caffeine on decreasing feeling of hunger was more potent than the CSD (p < 0.05 weeks 7-10 vs CSD) and kept it constant even during the follow up period. 

Scores obtained from subjects about their satisfaction, revealed that after week 4 they responded positively to the CSD treatment (p < 0.05 for weeks 4 and 5 and p < 0.01 for week 6) and this marked rate of satisfaction remained significant toward the rest of study even at follow-up period (p < 0.01 at first week of follow-up and p < 0.05 at three other weeks). Caffeine treatment along the CSD diet significantly increased CSD effect on satisfaction after week 8 (p < 0.05 vs. CSD group). 

During the study at CSD and follow-up stages, fullness remained unchanged compared to baseline valuein both groups, except for the two first weeks in which it decreased significantly (p < 0.05) in CSD group. 

## Discussion

In the present study a new format of diet was offered to obese and overweight subjects in which they were allowed four complete meals every day while preventing them from eating anything between meals. The concept of calorie shifting is that changing the amount of calorie consumed daily causes the body to respond by increasing its resting metabolic rate, meaning the it burns more calories. The theory is that by doing this, metabolism will not slow down as with many other diets and it will make losing weight easier. This is because, our metabolism automatically adjusts itself to run faster or slower based on the amount of food we have eaten over the past several days (18).

Caffeine treatment along the CSD regimen was done to increase the effect of calorie restriction on weight loss. The results of present study showed that weight loss by calorie shifting diet with caffeine occurred due to change in metabolism behavior in obese subjects which limited their energy intake to 1350 calorie per day. This pattern helped them approach a significant energy restriction (46% of basal value) reflected by obvious weight loss (8.25 Kg in 6 weeks) without being difficult for them to tolerate as demonstrated by VAS results (Figures 1-3). 

In addition in caffeine treated group the effect of CSD on weight and fat loss and VAS results was more potent and consistent even during the follow up period without regimen. It has been demonstrated that caffeine increases thermogenesis and fat oxidation through its inhibitory effects on cAMP degradation. In present study, RMR levels remained higher in caffeine treated group which reflected in higher weight and fat loss in this group and also increased fullness and satisfaction rate.

In a study by Klempel *et al.,* alternate day modified fasting was applied for weight loss in obese adults as a new strategy, in which all subjects consumed 25% of their baseline energy needs on the fast day (24 h), and then ate ad libitum on each alternating feed day (19). After 4 weeks of alternate day modified fasting body weight decreased by 5.6 Kg and interestingly subjects became habituated with alternate day modified fasting after approximately 2 weeks of diet by feeling less hunger during fast day. Decreased feeling of hunger was seen after four weeks in CSD in present study and showed that subjects could habituate to this system of diet with little effort. The advantage of CSD to alternate day modified fasting is that subjects were not require to endure intervals of fasting days.

On the other hand the satisfaction rate was increased from 31 mm at baseline to 53 on day 42 (after 6 weeks of CSD), that was similar to the results of alternate day modified fasting on satisfaction after 4 weeks of treatment (20). Insignificant effect of CSD on fullness both at treatment or follow-up period, also was the same as the results obtained from alternate day modified fasting on fullness after 8 weeks of study (19). Concomitant decrease of hunger and increase of satisfaction with diet within the short period of study (6 weeks) suggested that treatment was well-tolerated and obese subjects could effectively follow such diet for longer periods (21-23). All patients had been instructed to fill the VAS scales for each day at 11 p.m. and hence it might not show a clear profile of their hunger or satisfaction and fullness for the whole day. Therefore future studies with VAS evaluation during different time points in each day may help to have a more complete data set for these variables. 

In our knowledge we expected that Caffeine in CSD group, prevent the decline in RMR but we didn’t see this effect in our study. Although calorie restriction is frequently prescribed for obese subjects to facilitate weight loss (24, 25), adherence to regimen is always difficult for many obese patients (26, 27). Alternate-day fasting regimens and CSD can increase adherence to dietary restriction protocols because alternate-day fasting regimens require energy restriction only every other day and CSD regime restricts energy intake at specific intervals of each day without requiring for continuous fasting. It has been shown that adherence to the alternate-day fasting protocol was similar between the controlled food intake phase and the self-selected food intake phase which suggested that obese subjects are capable of self-selecting foods to meet their individual fast day energy goals (4). In the present study there was no significant difference in feeling hunger, satisfaction and fullness in CSD or follow-up period which highlighted the effectiveness of CSD regime on subjects habituation to the diet. Becauase during the follow up period, CSD+Caffeine treated group showed significant and consistent weight loss and VAS results compared to CSD group, it suggested that caffeine treatment has affected energy expenditure and uptake during this period. Subjects filled FFQ questionnaire for one month and a recall questionnaire for 5 days before diet to give enough information about their routine energy intake and dietary pattern. Consumption of calories on fast days was restricted to 1350 during CSD period. There was a direct correlation between calorie restriction due to CSD and weigh loss (p < 0.05; r=0.48). Reduced energy intake during 11 days of CSD was concomitant with non-significant reduction in RMR; however 3 days of higher calorie intake increased the mean level of RMR and prevented it from dropping out at consecutive days. The effect of short term energy restriction on RMR has been demonstrated in a study by Kouda K *et al.,* who showed that slight or moderate energy restriction (1462 kcal/day or 1114 kcal/day) for 4 days decreased basal metabolic rate (BMR) (28) which may justify the effects of 3 days of more calorie intake on preserving RMR at higher levels. After three phases of CSD (11-3; 11-3; 11-3 days), RMR remained statistically unchanged (1387 at day 42 compared to 1428 at baseline) and this is a valuable finding because the major limitation in most of calorie restriction diets is adaptation of metabolic system to new levels of energy intake, which decrease BMR and energy expenditure and leads to sustained body weight even at presence of low calorie intake (28). In addition to the shifting of diet during the study, the intake of each day was also restricted to four sessions that left 4 hours of intervals without intake. This feature decreased body weight and fat mass for the period of each 11 days, however assessing its efficacy in decreasing weight requires a study design which has a control group with same calorie intake without separating the meals into four parts. 

The effects of calorie restriction diets on decreasing coronary artery disease risk indicators in obese adults is an important aspect of studies carried out on weight loss. Total cholesterol concentrations decreased by 17.35% after 6 weeks of CSD diet and 14.66% after fallow-up period with self-selected diet. Triacylglycerol concentration was also decreased by 32.44% after 42 days of CSD regimen and 20.22% at the end of fallow-up period. These modulations in total-cholesterol and triacylglycerol concentrations have been shown previously in a study by Johnson *et al.,* in which they studied the effects of alternate day calorie restriction on clinical findings in overweight adults (29). Weight loss due to CSD regimen after 6 weeks were correlated to decreased levels of serum cholesterol and triacylglycerol which suggested that the degree of weight loss achieved by this CSD regimen may have contributed to the extent to which these plasma lipids were altered (30). Although the mean level of HDL-cholesterol concentrations increased after 6 weeks of CSD regimen, however the difference was not significant. While genetic factors are major determinants of high-density lipoprotein cholesterol (HDL-C), environmental factors also play a role. (31). This result is in accordance with the findings from the calorie restriction trials which had no effect on HDL cholesterol concentrations after short durations of treatment (32- 35).

The limitation of present study includes the absence of control group, because of our centers’ strategies and subject availabilities. Further studies which encompass a CSD and control group in parallel may reveal the effectiveness of CSD regimen in weight loss and subjects adherence to new diet. 

In conclusion CSD effectively reduced body weight and fat mass after 6 weeks of treatment, the effect that remained for at least one month. But caffeine treatment during and after CSD regimen produced constant weight loss profile even in follow-up period. RMR was not decreased during the CSD and suggested that high rate of metabolism is a key factor in continuous weight loss by CSD treatment. In addition both biochemical factors and patients satisfaction demonstrated CSD and caffeine potential applicability in clinic for obese subjects. Further studies with control groups will reveal the efficacy of CSD and caffeine as an alternative treatment for weight loss. 
